# Diphtheria in eastern Nepal.

**DOI:** 10.3201/eid0502.990225

**Published:** 1999

**Authors:** H Srinivasa, S C Parija, M P Upadhyaya


LHP had approximately 713,000 person-years of enrollment from 1993 to 1997. We identified 104 members with a *C. difficile* diagnosis on a claims record during this period. This group includes most of the patients in our original study. Most patients (62.5%) were identified exclusively from inpatient records; another 15.4% had both an inpatient and an outpatient record with a *C. difficile* diagnosis; and 22.1% had only an outpatient *C. difficile* diagnosis. We calculated an age-adjusted rate of infection (adjusted to the 1990 U.S. population), for each year and for the 5-year period. The incidence of *C. difficile* infection for all members during 1993 to 1997 was 14.8 cases per 100,000 person-years of enrollment. The patients rates for male and female were essentially the same (14.4 vs. 15.5, respectively). The rates increased dramatically with age. For persons ages 0-4, the age-adjusted rate per 100,000 person-years of enrollment (number of cases) was 5.3 (2); for 5-14, 2.7 (3), for 15-24, 2.2 (2); for 24-34, 6.4 (6); for 35-44, 9.2 (12); for 45-54, 15.7 (17); 55-64, 16.8 (10); 65-74, 38.5 (19); and 75+, 98.9 (33). The overall average rate of infection was 15.4; there were 104 cases.

The rate of infection may have declined since 1993 in this population. The 1993 rate was 24.5 per 100,000 person-years of enrollment, declining to 11.1 in 1997 (1993, 24.4; 1994, 19.1; 1995, 9.9; 1996, 12.3; and 1997, 11.1).

Our method for estimating rates has some limitations. We did not examine laboratory records to confirm the diagnosis. In addition, some laboratory-confirmed infections may not have resulted in a claims record with a *C. difficile* diagnosis. The Lovelace managed-care population is an insured, generally healthy population that may not have the characteristics of patients in other health care delivery settings or, because of its geographic restriction, the characteristics of the general U.S. population. Nevertheless, these estimates provide a basis for determining the magnitude of the public health problem of *C. difficile* infection. Additional surveillance studies are needed to better estimate the incidence of infection and to determine whether the incidence has declined during recent years.

**Floyd Frost,* Judith S. Hurley,***
**Hans V. Petersen,* and Roman N. Casciano†**
*Southwest Center for Managed Case Research, Albuquerque, New Mexico, USA; and †Analytica Group, Ltd., New York, New York, USA

### Reference

1. Frost F, Craun GF, Calderon RL. Increasing hospitalization and death possibly due to *Clostridium difficile* diarrheal disease. Emerg Infect Dis 1998;4:619-25.

### Diphtheria in Eastern Nepal

**To the Editor:** Diphtheria, caused by *Corynebacterium diphtheriae*, was a major childhood killer until the advent of the Expanded Program on Immunization when diphtheria, pertussis, and tetanus (DPT) vaccination was greatly increased; diphtheria gradually declined in many countries. We report two cases of diphtheria identified at the B.P. Koirala Institute of Health Sciences Hospital, Dharan, Nepal.

During April 1996, a 6-year-old patient had fever (for 5 days), difficulty in swallowing and breathing, and change of voice (for 4 days). Throat examination showed a grayish-white membrane over the right tonsil, uvula, and adjacent soft palate. The membrane could not be removed, and the larynx could not be examined. Swabs were taken from the membrane area and sent to the laboratory, where smears were made and stained by Gram and Albert stains. Gram-stained smears showed a large number of gram-positive bacilli with the appearance of Chinese letters, and Albert stain showed bacilli with numerous metachromatic granules. A diagnosis of faucial diphtheria, with a strong possibility of laryngeal diphtheria, was made. The patient was treated with parenteral penicillin and diphtheria antitoxin. His condition improved after 6 days of therapy.

In December 1996, a 9-year-old patient sought treatment for chronic pain and discharge in the left ear. On examination, he had mucopurulent discharge, antral perforation, and mastoid tenderness. Throat examination showed bilateral tonsilitis. A provisional diagnosis of acute mastoiditis associated with acute septic tonsillitis was made. Throat swabs were collected and sent to the laboratory; smear findings showed typical organisms morphologically resembling *C. diphtheriae*. Culture done on 10% defibrinated sheep blood agar and Loefflers serum slope grew colonies consistent with *C. diphtheriae*. In addition to local antibiotic to the ear, the patient was given parenteral penicillin, gentamicin, and metronidazole. Because the patient had no features of systemic toxicity, no antidiphtheria serum was administered. The patient became well and was discharged on day 4.

In the first case, a throat culture could not be done because the patient had already received local antiseptic paint. However, the diagnosis was clinically consistent with classic diphtheria with features of toxicity. In the second case, diphtheria was suspected only after bacteriologic examination. Unlike patient 1, patient 2 had no evident features of systemic toxicity. Hence the isolate could be nontoxigenic. Localized diphtheria due to nontoxigenic *C. diphtheriae* is known to occur (1).

The two patients did not give a complete history of immunization and may not have been vaccinated (or may have been partially vaccinated) with DPT. On the Indian subcontinent, DPT vaccination coverage is reported to be 80%. However, it may not be so in all areas, and immunization may have decreased to approximately 50% in certain areas of Southeast Asia (2). This may also be true in certain areas of eastern Nepal. An immunization status survey done in midwestern Nepal from 1989 to 1990 showed that DPT coverage was unsatisfactory (3). Lack of sustained immunization may even result in outbreaks. The recent epidemics of diphtheria in the Ukraine, Russian Federation, and other countries of the former Soviet Union are examples of resurgence due to ineffectively maintained immunization programs (4,5).

Diphtheria, still occasionally seen in many Southeast Asian countries including India and Nepal, is thought to be declining in these areas. However, accurate data have not been recently available, particularly from Nepal, because reporting is infrequent, laboratory confirmation is not available, and the extent of carriers is not clearly known (2).

These two cases show the persistence of diphtheria in a population in Nepal immunized with DPT and underscore the need for careful surveillance, laboratory documentation of clinical diphtheria, and increased immunization of children in this area.

**H. Srinivasa, S.C. Parija, and M.P. Upadhyaya**
B.P. Koirala Institute of Health Sciences,
Dharan, Nepal

### References

1. Dixon JMS, Noble WC, Smith GR. Diphtheria, other mycobacterial, and corynebacterial and coryneform infections. In: Topley and Wilson's principle of bacteriology, virology and immunology. Vol 3. 8th ed. London: Edwards Arnold; 1990. p. 55-79.
2. Srinivasa H. Immunizing children in Southeast Asia: a critical appraisal of current EPI status and future prospects. In: Immunizing agents for tropics: success, failure and some practical issues (BPKIHS Monograph Series 1). B.P. Koirala Institute of Health Sciences (BPKIHS) Hospital, Dharan, Nepal; 1997. p. 1-8.
3. Shrestha IB. Immunization status in mid-western region of Nepal. In: Health Research Abstract 1991-1994. Nepal Research Council 1995. Proceedings of Second National Seminar on Health Research in Nepal at Kathmandu, Nepal. 1994 Dec 20-22.
4. The World Health Reports. Fighting disease, fostering development. Report of the Director General, World Health Organization, Geneva; 1996.
5. Begg N, Balraj B. Diphtheria: are we ready for it? Archives of Childhood 1995:568-92.

### Commercial Use of *Burkholderia cepacia*

**To the Editor:** In their review of the potential threat to human health by the commercial use of *Burkholderia cepacia*, Holmes et al. (1) focus on the biopesticidal uses of this bacterium in agriculture. By virtue of its ability to antagonize a number of soilborne plant pathogens, *B. cepacia* is an attractive natural alternative to currently used chemical pesticides, such as captan, mancozeb, and metalaxyl. The replacement of these highly toxic agents, which are among the mainstays of crop protection chemicals, by safer products is a laudable goal. However, despite being nonpathogenic to healthy humans (and thus classified as a Biosafety Level 1 species), *B. cepacia* can cause life-threatening pulmonary infection in persons with cystic fibrosis. Holmes et al. call for a moratorium on the use of *B. cepacia* in agriculture until more is known about risks from such use.

Perhaps of greater concern than agricultural use is *B. cepacia*'s use as a bioremedial agent. Holmes et al. only briefly refer to the capacity of this species to degrade chlorinated aromatic substrates such as those found in certain pesticides and herbicides. By virtue of its extraordinary metabolic versatility, *B. cepacia* can use such compounds as nutrient carbon energy sources. In addition, some strains produce enzymes capable of degrading nonnutritive substrates, such as trichloroethyl-

